# ZnO nanoparticle-based seed priming modulates early growth and enhances physio-biochemical and metabolic profiles of fragrant rice against cadmium toxicity

**DOI:** 10.1186/s12951-021-00820-9

**Published:** 2021-03-17

**Authors:** Yuzhan Li, Luxin Liang, Wu Li, Umair Ashraf, Lin Ma, Xiangru Tang, Shenggang Pan, Hua Tian, Zhaowen Mo

**Affiliations:** 1 State Key Laboratory for Conservation and Utilization of Subtropical Agro-bioresources, College of Agriculture, South China Agricultural University, 510642 Guangzhou, China; 2 Crop Research Institute, Guangdong Academy of Agricultural Sciences, Guangdong Provincial Key Laboratory of Crop Genetic Improvement, Guangdong 510640 Guangzhou, China; 3 Department of Botany, Division of Science and Technology, University of Education, 54770 Lahore, Punjab Pakistan; 4 Scientific Observing and Experimental Station of Crop Cultivation in South China, Ministry of Agriculture and Rural Affairs, 510642 Guangzhou, China

**Keywords:** ZnO NPs, Cd, plant growth, physiological response, metabolomics

## Abstract

**Background:**

Cadmium (Cd) is amongst the most toxic heavy metals that severely affects crop growth, whereas application of nanoparticles (NPs) to negate the toxic effects of heavy metals could be an effective management approach. In the present study, the seeds of two fragrant rice varieties i.e., Yuxiangyouzhan and Xiangyaxiangzhan under normal and Cd stress conditions i.e., 0 and 100 mg L^− 1^ applied with four levels of ZnO NPs i.e., 0, 25, 50, and 100 mg L^− 1^.

**Results:**

Seed priming with ZnO NPs had no significant effect on the seed germination (*p* > 0.05) however, it substantially improved the seedling growth and other related physiological attributes under the Cd stress. The mean fresh weight of the shoot, and whole seedling was increased by 16.92–27.88% and by 16.92–27.88% after ZnO NPs application. The root fresh weight, root-shoot length was also substantially improved under ZnO NPs treatment. Moreover, application of ZnO NPs induced modulations in physiological and biochemical attributes e.g., the superoxide dismutase (SOD) activity in root and shoot, the peroxidase (POD) activity and metallothionein contents in root were increased under low levels of ZnO NPs. The α-amylase and total amylase activity were improved by ZnO NPs application under Cd Stress. Besides, modulation in Zn concentration and ZnO NPs uptake in the seedling were detected. The metabolomic analysis indicated that various pathways such as alanine, aspartate and glutamate metabolism, phenylpropanoid biosynthesis, and taurine and hypotaurine metabolism were possibly important for rice response to ZnO NPs and Cd.

**Conclusion:**

Overall, application of ZnO NPs substantially improved the early growth and related physio-biochemical attributes in rice. Our findings provide new insights regarding the effects of ZnO NPs on seed germination, and early growth of rice, and its potential applications in developing crop resilience against Cd contaminated soils.
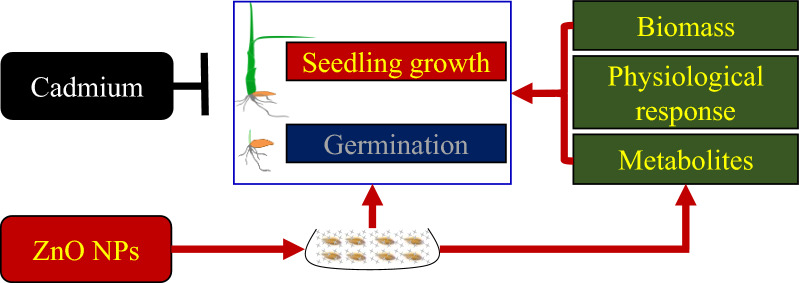

**Supplementary Information:**

The online version contains supplementary material available at 10.1186/s12951-021-00820-9.

## Background

Rice is the staple food for more than four billion people globally and it is cultivated worldwide across different climatic conditions. Among heavy metals, cadmium (Cd) is recognized as the most toxic heavy metal, and its concentration in arable lands has increased due to excessive use of agro-chemical and anthropogenic activities [1–3].

Rice generally has high bioaccumulation for various toxic metals such as arsenic (As), mercury (Hg), lead (Pb), and cadmium (Cd), respectively [4, 5]. Dietary intake of plant-derived foods that are rich in toxic metals especially As and Cd poses serious threats to consumers’ health [6]. Rice accounts for ~ 50% of the total Cd intake in people consuming rice as staple food [7]. Therefore, minimization of the transfer of Cd from the environment and/or rhizosphere to other plant parts specifically rice grains is important.

Although, Cd is a non-essential element, it is easily absorbed by plant roots, and competes with other bivalent ions such as Ca, Fe, Mn, and Zn to accumulate in plants [8–10], with subsequent phyto-toxic effects. The Cd toxicity in plants often leads to growth inhibition and disruption of physiological processes [11, 12]. Studies showed that Cd inhibits and reduces the germination rate, biomass accumulation, root-shoot ratio, and leaf development in rice [13, 14]. The inhibition in growth and biomass accumulation in rice are linked to the Cd-toxicity related mechanistic changes [15, 16]. Furthermore, Cd accumulation in rice plants can cause oxidative stress due to excess production of reactive oxygen species (ROS), and increased lipid peroxidation in plants [16, 17]. Plants produce enzymatic antioxidants such as superoxide dismutase (SOD), peroxidase (POD), and catalase (CAT) to protect themselves against oxidative stress induced by heavy toxic metals [17–19]. Additionally, hydrolyzing enzymes such as acid phosphatases, proteases, and α-amylases are known to facilitate both seed germination, as well as seedling growth by activating nutrients in the endosperm, however, in presence of heavy metals, starch might be immobilized thus limiting the nutrient sources [20]. Cd toxicity severely inhibits the germination index, vigor index, radicle length, and amylase activities in rice [21], therefore, it is imperative to improve the early growth of rice under Cd toxic conditions.

Recently, nanotechnology has been extensively employed in the field of plant sciences to explore its potential impacts in improving crop yields under metal toxic conditions [22, 23]. Due to high reactivity, large specific surface area, and strong adsorption capacity, nanoparticles (NPs) can adversely affect the transport of co-existing pollutants such as pesticides, heavy metals, and toxic organics [24–27]. Generally, the absorption and accumulation of heavy metals in plants are largely influenced by the application of NPs [28, 29], however, with the rapid development of nanotechnology, metal and metal oxide nanoparticle are being extensively used, and these NPs inevitably end up in soil or water and thus accumulate in the environment [30]. Due to their unique electronic, optical, dermatological, and antibacterial properties, the ZnO NPs are widely used in batteries, pigments, semiconductors, cosmetics, drug carriers, and other commercial products [31]. Scientists have different opinions regarding the positive or negative implications of NPs on crop plants, e.g., application of ZnO NPs has been reported to have negative effects which include inhibition of seed germination, reduction in biomass accumulation and chlorophyll content, and excessive production of ROS in plants [32, 33]. On the contrary, application of ZnO NPs at 50 ppm has been reported to improve the seedling growth and reduce the excessive generation of ROS whilst adverse effects on rice seedling growth have been observed at 500 and 1000 ppm concentrations [29]. Additionally, ZnO NPs could promote the rice growth at early stages and could alleviate the toxic effects of Cd [27]. Thus, NPs could have positive as well as negative effects on crop plants at the morphological, physiological, biochemical, and molecular levels, and this may depend on numerous factors like plant species, growth stage, growing conditions, application method, and application dose [34].

The ZnO NPs can be applied to the plants in different ways, however, seed priming is considered an easy method which may be beneficial in the early growth stages of plants in environment with adverse conditions [35]. Although, previous studies reported ZnO NPs-induced improvements in crop plants [28], the effects of seed priming with ZnO NPs under subsequent Cd stress in fragrant rice have not yet been explored. Therefore, the present study was conducted to assess the effect of ZnO NPs based seed priming on rice germination, growth, physiological response, and the changes in metabolites under subsequent exposure to Cd toxic conditions, with the hypothesis that application of ZnO NPs would improve the early growth of fragrant rice through ZnO NPs based seed priming. This study may find potential applications in improving the rice resilience against Cd contaminated conditions.

## Materials and methods

### Experimental materials

Seeds of two fragrant rice varieties i.e., Xiangyaxiangzhan and Yuxiangyouzhan, were obtained from College of Agriculture, South China Agricultural University, China. These two varieties are commercially popular in South China as fragrant rice varieties.

The ZnO NPs (with 99.9% purity, and particle size of 30 ± 10 nm) were purchased from Aladdin Bio-Chem Technology Co. Ltd., Shanghai, China. The characterization of the ZnO NPs was reported earlier in a previous study, the particles were described to have an average size of 36 nm, as assessed by scanning electron microscopy (SEM), and 28 nm as determined by transmission electron microscopy (TEM), matching the specifications mentioned by the manufacturer (~ 30 nm) [36].

### Experimental details

The experiments were conducted at the College of Agriculture, South China Agricultural University, Guangzhou, China. The seeds of the two rice cultivars i.e., Xiangyaxiangzhan and Yuxiangyouzhan were treated with four ZnO NPs treatments at 0, 25, 50 and 100 mg L^− 1^, respectively and then exposed to two Cd levels i.e., 0 and 100 mg L^− 1^. The concentrations of ZnO NPs and Cd used in this study were based on previous reports [28, 29, 36, 37], which suggested that low dose of ZnO NPs (0–100 mg L^−1^) may have positive effect of plant growth whilst the Cd treatment with 100 mg L^−1^ could lead to germination and growth inhibition. There were 16 treatments in total and each treatment has 6 repeats with total 96 glass petri dishes in total.

The seeds of both rice varieties were surface sterilized with 5% sodium hypochlorite solution for 15 min, and then rinsed thoroughly with deionized water. The different concentration of ZnO NPs were prepared by weighing different amounts of ZnO NPs, the ZnO NPs were added to deionized water (pH = 6.5) and stirred for 5 min, then followed by their dispersion in deionized water by water bath with ultrasonic treatment for 30 min [36]. The seeds were then soaked in the prepared ZnO NPs solutions for 20 h at 25 ℃ in the dark with continuous aeration, and then rinsed three times with deionized water. The change of pH and Zn^2+^ in the ZnO NPs solutions and the Zn concentration in the seed after priming were detected (Additional file 1: Figure S1). Zhang et al. [38] reported that the ZnO NPs dissolution in water (pH = 7.5) with Zn^2+^ ions of about 1.2 mg L^−1^. In this study, the ZnO NPs dissolved in water (pH = 6.0–6.5) with a low concentration of Zn ions is obvious (the dissolution of the ions is about 1.2–1.5 mg L^−1^) (Additional file 1: Figure S1). Study have reported that seed primed with 2.5 mM of Zn resulted in increased seed Zn content and seedling growth in rice [39].

Total 100 seeds were allowed to germinate on two layers of filter paper placed in a 150 mm diameter glass petri dishes. The filter paper in petri dishes were keep wet condition by covered with approximately 20 ml of Cd solution. The solution in the petri dishes was changed daily by replacing with fresh Cd solution in order to keep the concentration of the Cd constant. The petri dishes were placed in an incubator with a 14 h/28 ℃ day, and a 10 h/25 ℃ night, photosynthetically active radiation with a light intensity of 400 µmol of photons m^− 2^ s^−1^ at daytime was maintained, and the relative humidity was set at 70%.

### Sampling and measurements

The germination was monitored every day after sowing, and the morphological traits were recorded from 4 randomly selected glass petri dishes in each treatment at seven days after sowing. Sample of 60 seedlings were randomly selected from each of the 4 randomly selected glass petri dishes of each treatment, and then placed immediately in liquid nitrogen, and stored at − 80 °C for subsequent assessment of physiological and metabolites profiles.

#### Determination of germination traits

Germination traits of seeds were recorded daily after the radicle length exceeded 2 mm. The germination rate is the ratio of the number of germinated seeds to the total number of seeds sown and is expressed as a percentage. Seed vigor index was calculated by multiplying the germination index with the shoot length as described previously [40].

#### Determination of morphological traits

Ten seedlings from each of four randomly selected glass petri dishes were randomly harvested at seven days after sowing, and the length of the prophyll, leaf, leaf sheath, leaf blade, root, and shoot were measured. Another ten seedlings from each of four randomly selected glass petri dishes were randomly harvested for measurement of the fresh weight of the root, shoot, and seedling by using the electronic balance (G&G Measurement Plant, Suzhou, Jiangsu, China).

#### Determination of antioxidant enzyme activities and malondialdehyde contents

The antioxidant enzyme activities and malondialdehyde (MDA) content were quantified according to method reported previously [41]. Fresh samples of germinated seedlings (0.3 g) were homogenized with 3 ml of 100 mM phosphate-buffered saline (PBS) solution, centrifuged at 14000 × *g* for 15 min at 4 °C. The supernatant consisted of the crude enzyme extract which was subjected to subsequent assays for the determination of the activities of antioxidants and the MDA content.

For superoxide dismutase (SOD) activity, crude enzyme extract (0.05 ml) was mixed with 1.75 ml of sodium phosphate buffer (pH = 7.8), 0.3 ml of 130 mM methionine buffer, 0.3 ml of 750 µmol nitroblue tetrazolium (NBT) buffer, 0.3 ml of 100 µmol EDTA-Na_2_ buffer, and 0.3 ml of 20 µmol lactoflavin in 5 ml beaker, and allowed to react under 4000 l × light for 30 min. The absorbance of reacted solution was recorded at 560 nm by using a benchtop spectrophotometer (BioTek Instruments Inc; Winorsky, Vermont, USA). One SOD active unit (U) is defined as the amount of the enzyme that inhibits 50% photochemical reduction of substrate NBT, and SOD activity was defined in the unit U g^−1^ fresh weight (FW) min^−1^.

For peroxidase (POD) activity, 0.05 ml crude enzyme extract was mixed with 1 ml of 0.3% H_2_O_2_, 0.95 ml of 0.2% guaiacol, and 1 ml of 50 mM sodium phosphate buffer (pH = 7.0). The absorbance was recorded at 470 nm by using a benchtop spectrophotometer at an interval of 30 s, with 5 replicates. One POD active unit (U) was defined as the amount of enzyme that catalyzed the reaction such that there was a reduction of 1 absorbance per min, and the POD activity was defined as U g^−1^ FW min^−1^.

For catalase (CAT) activity, 0.05 ml crude enzyme extract was mixed with 1 ml of 0.3% H_2_O_2_, and 1.95 ml of sodium phosphate buffer. The absorbance was recorded at 240 nm every 30 s, with 4 replicates. One CAT active unit (U) was defined the amount of enzyme that catalyzed the reaction such that there was a reduction of 0.1 absorbance per min, and the CAT activity was defined as U g^−1^ FW min^−1^.

For assessing the MDA content, crude extract was reacted with thiobarbituric acid (TBA) in a boiling water bath for 30 min, and the absorbance of the supernatant was recorded at 532, 600, and 450 nm, respectively using a benchtop spectrophotometer, and the MDA content was expressed as µmol g^−1^ FW.

#### Determination of the amylase enzyme activity

The amylase enzyme activity was estimated as previously described [42]. Samples of fresh germinated seedling (0.3 g) were homogenized in liquid N_2_ with mortar and pestle along with 3.75 ml citrate buffer solution (pH = 5.6), and the homogenized contents were transferred to a 10 ml centrifuge tube and subjected to centrifugation at 12000 × *g* for 10 min at 4 ℃. The supernatant was transferred to a 25 ml volumetric flask, and diluted with deionized water to 25 ml, and mixed thoroughly. This enzyme extract (1 ml) was pipetted into a 10 ml centrifuge tube. The subsequent steps were the same for quantification of α-amylase activity as well as the total amylase activity, with the exception that the enzyme for α-amylase activity needed to be heated in water bath at 70 ℃ for 15 min. The enzyme extract (1 ml) and citrate buffer solution (pH = 5.6, 1 ml) were pipette into a 10 ml centrifuge tube and heated in water bath at 40 ℃. After 15 min of heating, 2 ml of 1% soluble starch solution was added into the tube, followed by the addition of 4 ml of 4 mol L^−1^ NaOH solution to end the reaction. The reaction solution (1 ml) as well as 1 ml of 3,5-dinitrosalicylic acid were added to a 10 ml centrifuge tube, and heated in boiling water for 5 min, and then cooled to room temperature, and diluted with 8 ml deionized water. The absorbance was recorded at 520 nm by using a benchtop spectrophotometer. The amylase activity was expressed as mg g^−1^ FW min^−1^. The β-amylase activity value was equal to α-amylase activity value subtracted out of the total amylase activity value.

#### Determination of Metallothionein (MT)

Samples of fresh germinated seedlings (0.3 g) were crushed in liquid N_2_ with mortar and pestle, and homogenized with 1 ml of buffer (20 µmol Tris-HCl (pH = 8.6), and 0.5 µmol phenylmethylsulfonyl fluoride), and 3 ml of 0.01% β-mercaptoethanol, and the resulting sample was transferred to a 10 ml centrifuge tube, and centrifuged at 10000 × *g* for 60 min at 4 °C. The supernatant was pipetted into a 10 ml centrifuge tube, and heated at 70 ℃ in water bath for 10 min, and centrifuged at 10000 × *g* for 60 min at 4 °C. The supernatant was the MT extract [43], and the MT quantification was performed according to Ellman [44]. The MT extract (0.2 ml), 4.4 ml of mixture solution (4.4 mmol HCl and 1.76 µmol EDTA), and 0.4 ml of 0.04% 5,5-dithio-bis-(2-nitrobenzoic acid) (DTNB) containing 0.8 mmol NaCl were pipetted into a 5 ml beaker, and placed in dark for 30 min. The absorbance of the reaction solution was recorded at 412 nm by using a benchtop spectrophotometer. The concentration of MT was calculated according to method described previously [45].

#### Determination of chlorophyll contents

Fresh shoot samples (0.1 g) were extracted with 6 ml of 95% ethanol and put in the dark place for at 4 ℃. After 24 h, the samples were centrifuged at 700 × *g* for 10 min at 4 ℃. The absorbance of the supernatant was recorded at 665, 649 and 470 nm and the chlorophyll and carotenoids content were estimated [46].

#### Determination of Zn and Cd concentration

Dry seedling and seeds samples of 0.50 g were digested with 12 ml HNO_3_:HClO_4_ (4:1 v/v) and resultant solutions were diluted to 25 ml and then filtered. The Zn ^2+^ in the solution was measured after filtration. The Zn and Cd content were determined using the Atomic Absorption Spectrophotometer (AA6300C, Shimadzu, Japan) [19].

#### Transmission electron microscopy (TEM) observation and element analysis

Fresh roots and shoots of germinated seedlings were thoroughly washed with deionized water, and then immersed in PBS (0.1 m) for 20 min, then centrifuge and pour off the supernatant, repeat three times, soaked with 4 ℃ osmic acid (1%) for 3 h, then immersed in PBS (0.1 m) for 20 min, centrifuged to remove the supernatant, then soaked with a series of gradient alcohol (30, 50, 70, 80, 85, 90, 95, 100%) for 15 min, immersed in acetone: epoxy resin (2:1), acetone: epoxy resin (1:1) and epoxy resin with 37 ℃ for 12 h, respectively. And then put the sample in a small capsule, added epoxy and waited for 48 h at 60 ℃, and sliced on ultra-thin slicer (EM UC7, Leica, Germany). After staining with lead and uranium, transmission electron microscopy (Tecnai G2 20 TWIN, FEI, USA) was performed [47]. To further analyzed the existence of ZnO nanoparticles in the plant tissues, the Scanning Electron Microscope (SEM, Zeiss Sigma 300, England) and Energy dispersive X-ray spectroscopy (EDS, XFlash6I30, USA) are performed.

#### Metabolite extraction and metabolite profiling

Fresh shoot samples (1.0 g) were cut from rice seedlings, washed with deionized water, and snap-frozen in liquid N_2_ for 10 min, and stored at − 80 ℃ in triplicate. The metabolite extraction and metabolite profiling analysis were performed at Wuhan MetWare Biotechnology Co., Ltd., China (www.metware.cn) following their standard procedures. In brief, the shoot was crushed using a mixer mill. The samples in their powdered form were weighed (100 mg), and extracted overnight in 0.6 ml of 70% aqueous methanol at 4 ℃ followed by centrifugation at 10000 × *g* for 10 min. The extract was absorbed using a CNWBOND Carbon-GCB SPE Cartridge (250 mg, 3 ml; ANPEL, Shanghai, China,), and filtered using a 0.22 µm pore size, and analyzed using an LC-ESI-MS/MS system (HPLC, Shim-pack UFLC SHI-MADZU CBM30A system, ; MS, Applied Biosytems 4500 Q TRAP). The mobile phase consisted of pure water with 0.04% acetic acid (solvent A), and acetonitrile with 0.04% acetic acid (solvent B). The sample measurements were performed with a gradient program that employed starting conditions of 95% A and 5% B. A linear gradient to 5% A and 95% B was programmed to be achieved within the first 10 min, and this level was maintained for 1 min and then, a composition of 95% A and 5% B was achieved within a rapid time frame of 0.1 min which was maintained for 2.9 min. The column oven was set to 40 °C, and the injected volume was 4 µl. The effluent was alternatively connected to an ESI-triple quadrupole-linear ion trap (QTRAP)-MS. On a triple quadrupole-linear ion trap mass spectrometer (Q TRAP), LIT and triple quadrupole (QQQ) scans were performed. The instrument was equipped with an ESI Turbo Ion-Spray interface, and the API 4500 Q TRAP UPLC/MS/MS System was controlled by Analyst 1.6.3 software (AB Sciex).

### Statistical analyses

Analysis of Variance (ANOVA) and correlation analyses were performed by using Statistix version 8 (Tallahassee, Florida, USA) followed by comparisons of the sample means using Tukey’s HSD test at *p* < 0.05 level. For multivariate analysis, data were imported into the MetaboAnalyst software (), and the heat map for the investigated parameters was established. The principal component analysis (PCA) and partial least squares-discriminant analysis (PLS-DA) were performed to examine the intrinsic variation in the parameters, and to reduce the dimensionality of the data [48].

## Results

### The germination and seed vigor index

**Fig. 1:**
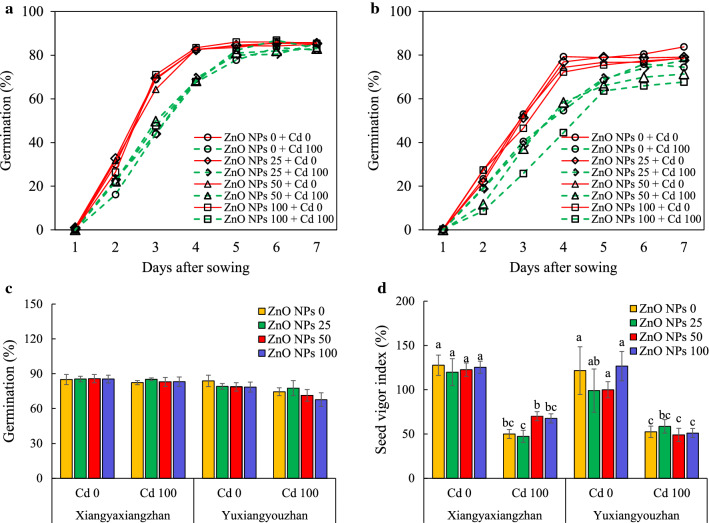
Effect of ZnO NPs on the dynamic of seed germination. **a** Xiangyaxiangzhan and **b** Yuxiangyouzhan, germination (**c**), seed vigor index (**d**) under Cd stress. ZnO NPs 0, ZnO NPs 25, ZnO NPs 50 and ZnO NPs 100: 0, 25, 50 and 100 mg L^−1^ of ZnO NPs. Cd 0 and Cd 100: 0 and 100 mg L^−1^. Values were represented as mean ± SD (n = 4). Different low case letters among the treatments within a variety shows the statistically significant at *p* < 0.05 according to least significant different test

Statistically significant differences (*p* < 0.05) were noted across varieties (V), and Cd treatments, while differential doses of ZnO NPs treatments did not induce any significant difference in seed germination in both rice varieties. The germination dynamics showed that Cd treatment reduced the germination of rice seeds particularly at 3–5 days after sowing. Cd and V × ZnO NPs × Cd markedly affected seed vigor index. On an average, Cd stress decreased the germination rate, and the vigor index by 5.59% and 52.68%, respectively, as compared with no Cd treatment (Fig. 1, Additional file 2: Table S1).

### Morphological growth attributes

**Fig. 2:**
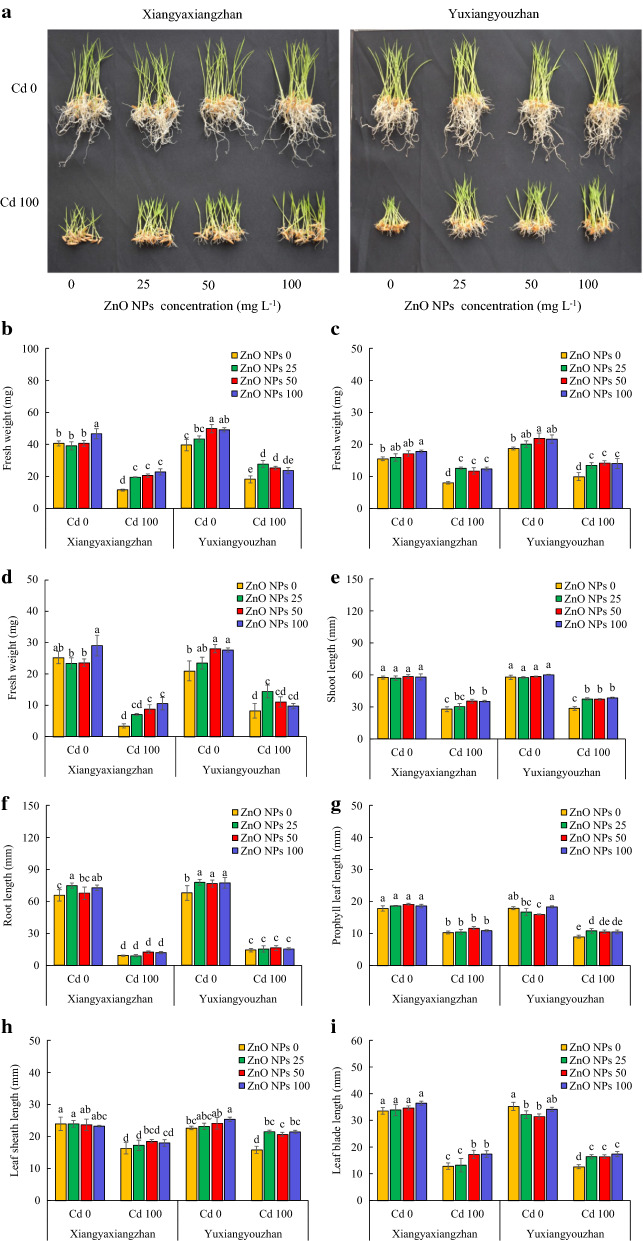
The growth photographs and fresh weight of rice seedlings. Growth photographs (**a**), total fresh weight (**b**), shoot fresh weight (**c**), root fresh weight (**d**), shoot length (**e**), root length (**f**), prophyll leaf length (**g**), leaf sheath length (**h**), and leaf blade length (**i**) of rice seedlings. ZnO NPs 0, ZnO NPs 25, ZnO NPs 50 and ZnO NPs 100: 0, 25, 50 and 100 mg L^−1^ of ZnO NPs. Cd 0 and Cd 100: 0 and 100 mg L^−1^. Values were represented as mean ± SD (n = 4). Different low case letters among the treatments within a variety shows the statistically significant at *p* < 0.05 according to least significant different test

Cd exposure revealed signs of over-stress, while seeds treated with ZnO NPs showed improvement in the growth of rice seedlings under Cd treatment (Fig. 2a). The fresh weight of the seedlings was significantly affected by variety, Cd, ZnO NPs, V × Cd, and ZnO NPs × Cd (Additional file 2: Table S1). The total fresh weight of rice seedlings substantially improved under ZnO NPs treatment (100 mg L^−1^) for Xiangyaxiangzhan, and at ZnO NPs doses of 50 and 100 mg L^−1^_,_ respectively for Yuxiangyouzhan under no Cd treatment. ZnO NPs treatments increased the total fresh weight of seedling in Xiangyaxiangzhan by 70.74, 79.48, and 99.56% at treatment of 25, 50, and 100 mg L^−1^, respectively, when exposed to Cd. Similarly, the total fresh weight of seedling in Yuxiangyouzhan markedly increased by 52.92% and 39.16% at 25, 50, and 100 mg L^−1^ ZnO NPs, respectively (Fig. 2b). The shoot fresh weight of rice seedlings significantly increased at ZnO NPs at 100 mg L^−1^ for Xiangyaxiangzhan, and 50 mg L^−1^ for Yuxiangyouzhan without Cd. The ZnO NPs treatments significantly increased the shoot fresh weight of seedlings in Xiangyaxiangzhan by 55.90, 45.96, and 53.42% at 25, 50, and 100 mg L^−1^ of ZnO NPs, respectively. Regarding Yuxiangyouzhan, the shoot fresh weight was increased by 35.18%, 43.22%, and 41.21% at 25, 50, and 100 mg L^−1^ ZnO NPs, respectively under Cd toxic conditions (Fig. 2c). Under normal conditions, the ZnO NPs application at 50 and 100 mg L^−1^ significantly increased the root fresh weight in Yuxiangyouzhan. The fresh root weight significantly increased in ZnO NPs treatment i.e., 50 and 100 mg L^−1^ for Xiangyaxiangzhan, while fresh root weight was enhanced in Yuxiangyouzhan at 25 mg L^−1^ of ZnO NPs (Fig. 2d).

The length of shoot, root, prophyll, leaf, leaf sheath, and leaf blade were significantly affected by Cd, ZnO NPs, and ZnO NPs × Cd (Additional file 2: Table S1). No significant variation was noticed in the shoot length of ZnO NPs treated rice seedlings under non-stressed seedlings. The shoot length of rice seedlings of both varieties improved by ZnO NPs treatment under Cd exposure (100 mg L^−1^). The shoot length of rice seedlings improved significantly in Xiangyaxiangzhan exposed to ZnO NPs application at 50 mg L^−1^ (27.20%), and 100 mg L^−1^ (26.84%). In Yuxiangyouzhan, the shoot length was increased by 29.90–34.79% under the different ZnO NPs treatment (Fig. 2e). Moreover, the root length of rice seedlings significantly increased in Xiangyaxiangzhan at 25 and 100 mg L^−1^ ZnO NPs, respectively. In Yuxiangyouzhan, all ZnO NPs treatments under normal conditions exhibited increase in root length of rice seedlings, while the ZnO NPs-based treatments did not influence the root length under Cd stress (Fig. 2f). The prophyll leaf length decreased significantly in Yuxiangyouzhan with and without Cd stress under ZnO NPs application at 25 and 50 mg L^−1^, respectively (Fig. 2g). ZnO NPs treatment at 100 mg L^−1^ led to substantial improvements in the leaf sheath length in Yuxiangyouzhan under normal conditions, while all ZnO NPs treatment significantly increased the leaf sheath under Cd stress (Fig. 2h). The leaf blade length in Yuxiangyouzhan was significantly decreased at ZnO NPs treatment at 25 and 50 mg L^−1^, respectively under normal conditions. In Xiangyaxiangzhan under Cd stress, the leaf blade length improved when treated with 50 and 100 mg L^−1^ ZnO NPs, while similar effects were noticed in Yuxiangyouzhan under all ZnO NPs treatment (Fig. 2i).

### Physiological responses of rice seedlings

#### Activity of amylase enzymes

**Fig. 3:**
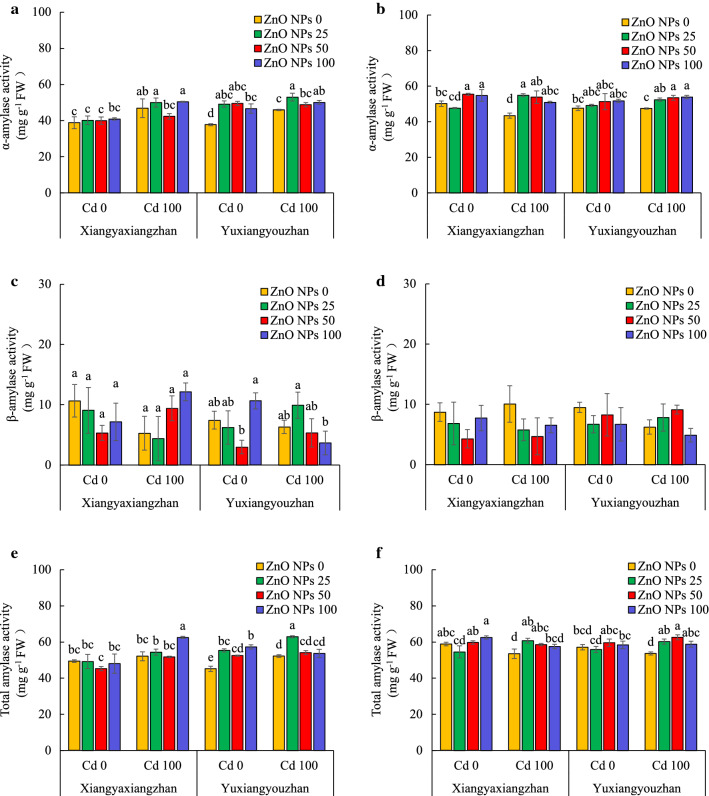
The α-amylase, β-amylase and total amylase activity of rice seedlings. α-amylase activity in shoot (**a**), α-amylase activity in the seedling (**b**), β-amylase activity in shoot (**c**), β-amylase activity in the seedling (**d**), total amylase activity in shoot (**e**), total amylase activity in the seedling (**f**). ZnO NPs 0, ZnO NPs 25, ZnO NPs 50 and ZnO NPs 100: 0, 25, 50 and 100 mg L^−1^ of ZnO NPs. Cd 0 and Cd 100: 0 and 100 mg L^−1^. Values were represented as mean ± SD (n = 4). Different low case letters among the treatments within a variety shows the statistically significant at *p* < 0.05 according to least significant different test

The activity of α-amylase and total amylase was significantly affected by ZnO NPs and Cd applications. The ZnO NPs × Cd significantly affected the activity of α and β-amylase (in seed), and total amylase (in the seedling) (Additional file 2: Table S1). For Yuxiangyouzhan, the activity of α-amylase in the seed was increased up to 30.29% after exposure to differential dosages of ZnO NPs treatments under normal conditions, whilst the activity of α-amylase in the seed under Cd stress (100 mg L^− 1^) significantly increased after exposure to ZnO NPs treatment at 25, and 100 mg L^−1^, respectively. The activity of α-amylase in Xiangyaxiangzhan rice seedlings increased after ZnO NPs treatment at concentrations of 50 and 100 mg L^−1^, respectively under normal conditions. The α-amylase activity in Cd stressed seedlings significantly increased by 17.45%-26.56% and 10.56%-13.71% in both rice varieties under all ZnO NPs treatments (Fig. 3a and b). The activity of β-amylase was not significantly affected by ZnO NPs treatments with and without Cd stress (Fig. 3c and d). The highest activity of total amylase in the seeds of Xiangyaxiangzhan and Yuxiangyouzhan was detected at 25 and 100 mg L^−1^ of ZnO NPs, respectively under Cd exposure. Under normal conditions, the activity of total amylase in the seed of Yuxiangyouzhan significantly increased by 24.23, 16.20, and 26.88% at 25, 50 and 100 mg L^−1^ ZnO NPs. In addition, under Cd exposure, the total amylase activity increased in Xiangyaxiangzhan and Yuxiangyouzhan by 7.53–13.57% and 9.58–16,64%, respectively, and statistically significant change was detected for all the ZnO NPs treatments, with the exception of 100 mg L^−1^ ZnO NPs in Xiangyaxiangzhan (Fig. 3e and f).

#### Activities of antioxidant enzymes

**Fig. 4:**
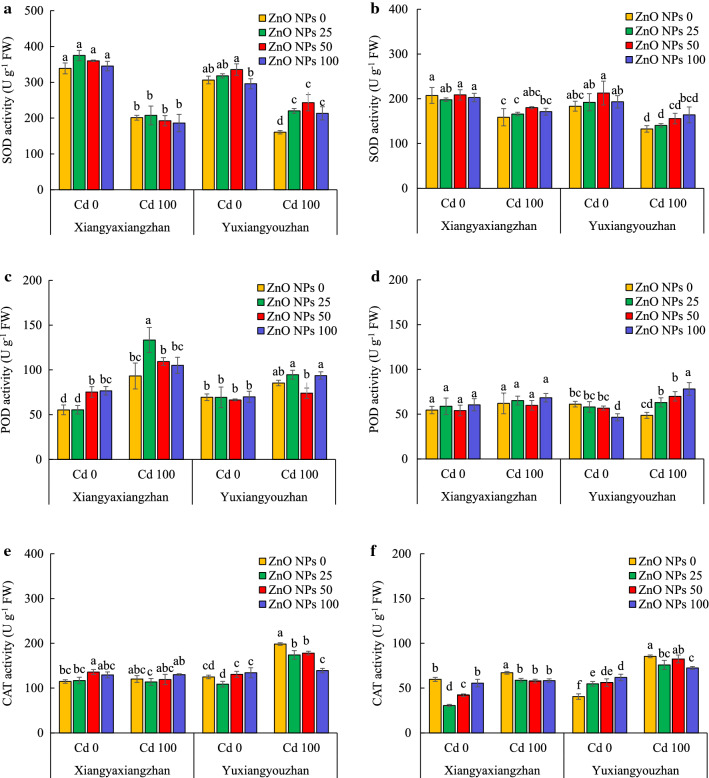
The antioxidant enzyme activities of rice seedlings. SOD activity in shoot (**a**), SOD activity in the seedling (**b**), POD activity in shoot (**c**), POD activity in the seedling (**d**), CAT activity in shoot (**e**), CAT activity in the seedling (**f**). ZnO NPs 0, ZnO NPs 25, ZnO NPs 50 and ZnO NPs 100: 0, 25, 50 and 100 mg L^−1^ of ZnO NPs. Cd 0 and Cd 100: 0 and 100 mg L^−1^. Values were represented as mean ± SD (n = 4). Different low case letters among the treatments within a variety shows the statistically significant at *p* < 0.05 according to least significant different test

The activity of SOD, POD, and CAT in the shoot, and the seedlings was significantly affected by Cd and ZnO NPs. The ZnO NPs × Cd significantly influenced the POD and the CAT in the shoot as well as in the total seedling (Additional file 2: Table S1). The activity of SOD in the shoot of Yuxiangyouzhan was increased by 36.99, 51.37%, and 32.79 at 25, 50, and 100 mg L^−1^ ZnO NPs, respectively under Cd stress. No significant changes were observed in shoot SOD activity in Xiangyaxiangzhan for the ZnO NPs treated groups under normal and Cd stress. The SOD activity was marginally increased in rice seedlings under Cd exposure, while no significant changes were observed for the ZnO NPs treated groups under normal and Cd stress conditions (Fig. 4a and b). The POD activity in the shoot of rice seedlings increased significantly by 36.23 and 38.56% at 50 and 100 mg L^−1^ ZnO NPs, respectively, without Cd. ZnO NPs at 25 mg L^−1^ increased the activity of POD in the shoot of rice seedlings by 43.06% under Cd exposure. Without Cd stress, ZnO NPs at 100 mg L^−1^ significantly decreased the POD activity. The POD activity in the seedlings of Yuxiangyouzhan increased by 29.61, 43.05, and 61.97% at 25, 50, and 100 mg L^−1^ ZnO NPs, respectively under Cd stress. No significant changes were observed in the POD activity in the shoot of Yuxiangyouzhan, and the seedling of Xiangyaxiangzhan in ZnO NPs treated groups under normal and Cd stress conditions (Fig. 4c and d). In the shoot of Xiangyaxiangzhan, the CAT activity significantly increased under normal conditions at 50 mg L^−1^ ZnO NPs, while a significant decrease in all the ZnO NPs treated groups was observed when exposed to Cd stress. The ZnO NPs treatment at 25 and 50 mg L^−1^ significantly decreased the CAT activity in the seedling of Xiangyaxiangzhan, while it increased in the seedlings of Yuxiangyouzhan under non-stressed conditions. The ZnO NPs treatments resulted in reduced CAT activity in the seedlings, while significant reduction was detected for Xiangyaxiangzhan in all the ZnO NPs treated groups, and for Yuxiangyouzhan in groups exposed to 25 and 100 mg L^−1^ of ZnO NPs (Fig. 4e and f).

#### The MDA contents and MT concentration

**Fig. 5:**
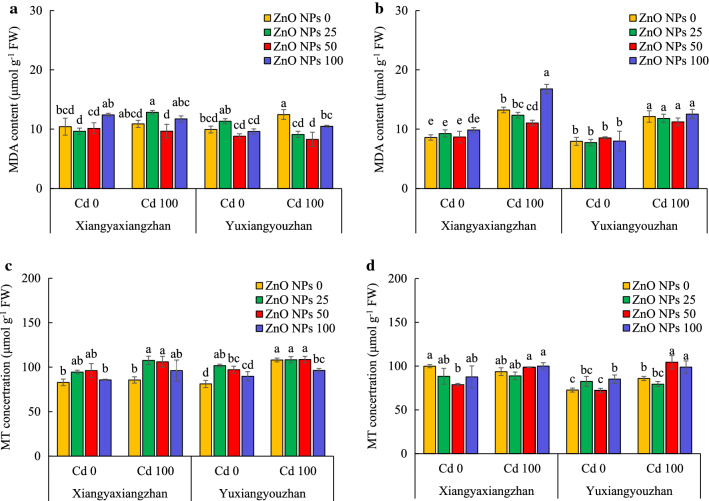
The MDA content and MT concentration of rice seedlings. MDA content in shoot (**a**), MDA content in the seedling (**b**), MT concentration in shoot (**c**), and MT concentration in the seedling (**d**). ZnO NPs 0, ZnO NPs 25, ZnO NPs 50 and ZnO NPs 100: 0, 25, 50 and 100 mg L^−1^ of ZnO NPs. Cd 0 and Cd 100: 0 and 100 mg L^−1^. Values were represented as mean ± SD (n = 4). Different low case letters among the treatments within a variety shows the statistically significant at *p* < 0.05 according to least significant different test

The MDA content and the concentration of MT were significantly affected by V, Cd, ZnO NPs, and ZnO NPs × Cd (Additional file 2: Table S1). The MDA content in shoot was not significantly affected by ZnO NPs treatments for both Xiangyaxiangzhan (with or without Cd stress) and Yuxiangyouzhan (exposed to 100 mg L^− 1^ Cd). The MDA content was decreased by 26.94, 33.60, and 15.88% at ZnO NPs dosages of 25, 50, and 100 mg L^− 1^, respectively, under Cd stress. The MDA content in the seedling was not significantly affected by ZnO NPs treatments for Xiangyaxiangzhan (in absence of Cd), and Yuxiangyouzhan (with or without Cd stress). The MDA content reduced by 16.63% and 26.83 at ZnO NPs at 50 and 100 mg L^− 1^, respectively as compared to untreated control (0 mg L^− 1^ ZnO NPs) under Cd exposure (Fig. 5a and b). The concentration of MT in the shoot of Xiangyaxiangzhan was increased by 25.58 and 23.90%, at 25, and 50 mg L^− 1^ ZnO NPs treatments, respectively, while ZnO NPs treatment at 100 mg L^− 1^ substantially reduced the shoot MT concentration of Yuxiangyouzhan as compared to untreated control (0 mg L^− 1^ ZnO NPs) under Cd stress. Moreover, the MT concentration in the shoot of Yuxiangyouzhan was increased by 25.43 and 19.68%, at 25, and 50 mg L^− 1^ ZnO NPs, respectively, under non-stress conditions. The ZnO NPs at 50 mg L^− 1^ exhibited significant decrease in MT concentration in the seedlings of Xiangyaxiangzhan under non-stressed conditions, however, the concentration of MT in seedlings of Yuxiangyouzhan significantly increased at 100 mg L^− 1^ ZnO NPs. Moreover, the concentration of MT in seedling of Yuxiangyouzhan was increased by 21.77 and 15.18% at 50 and 100 mg L^− 1^ ZnO NPs dosages, respectively under Cd exposure (Fig. 5c and d).

#### The chlorophyll contents

The chl a, chl b, total chl content, and carotenoids in shoot were significantly affected by V, Cd, ZnO NPs, and ZnO NPs × Cd (Additional file 2: Table S1). The ZnO NPs treatments reduced the chl a, chl b, total chl content, and carotenoids in shoot for Xiangyaxiangzhan under both normal and Cd stress conditions and for Yuxiangyouzhan under non-stress conditions only. Under Cd exposure, the ZnO NPs treatments improved the chl a, chl b, total chl content, and carotenoids in shoot of Yuxiangyouzhan (Additional file 1: Figure S2).

### The concentrations of Zn and Cd and the TEM images

**Fig. 6:**
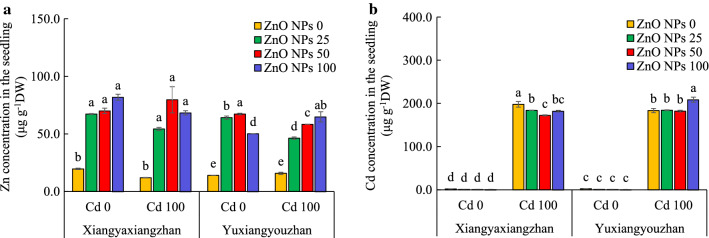
The Zn and Cd concentration of rice seedlings. Zn concentration in the seedling (**a**) and Cd concentration in the seedling (**b**). ZnO NPs 0, ZnO NPs 25, ZnO NPs 50 and ZnO NPs 100: 0, 25, 50 and 100 mg L^−1^ of ZnO NPs. Cd 0 and Cd 100: 0 and 100 mg L^− 1^. Values were represented as mean ± SD (n = 3). Different low case letters among the treatments within a variety shows the statistically significant at *p* < 0.05 according to least significant different test

The Zn and Cd concentration in the seedling were significantly affected by V, ZnO NPs, ZnO NPs × Cd, and V × ZnO NPs × Cd. Application of Cd significantly enhanced the Cd contents in the seedlings of both rice cultivars (Additional file 2: Table S1). The Zn concentration under ZnO NPs treatments was significantly higher than non-ZnO NPs treatment for both rice varieties, whilst the Cd level under ZnO NPs treatments reduced significantly for Xiangyaxingzhan. The concentration of Cd in seedling of Yuxiangyouzhan increased significantly at 100 mg L^− 1^ ZnO NPs under Cd exposure (Fig. 6). Further, the TEM images of root and shoot tissue in the germinated seedlings showed that the Zn level increased and positively associated with the external ZnO NPs concentrations as the ZnO NPs was transferred from the seeds to the seedlings during seed germination and then the DES analysis confirmed the element (Additional file 1: Figure S3).

### Metabolite analysis

#### Description of the metabolites

Among four ZnO NPs treatment, 50 mg L^− 1^ ZnO NPs treatment had the substantial alleviatory effects for both rice varieties under Cd stress. The shoot of Xiangyaxiangzhan and Yuxiangyouzhan under four treatments (A: ZnO NPs 0 + Cd, B: ZnO NPs 0 + Cd 100, C: ZnO NPs 50 + Cd 0, and D: ZnO NPs 50 + Cd 100) were sampled for analysis of metabolites (Additional file 3: Table S2). A total of 338 metabolites were detected including 72 amino acids and derivatives, 97 phenolic acids, 57 lipids, 44 organic acids, 19 saccharides and alcohols, 9 vitamin and 40 nucleotides and derivatives (Additional file 3: Table S2).

**Fig. 7:**
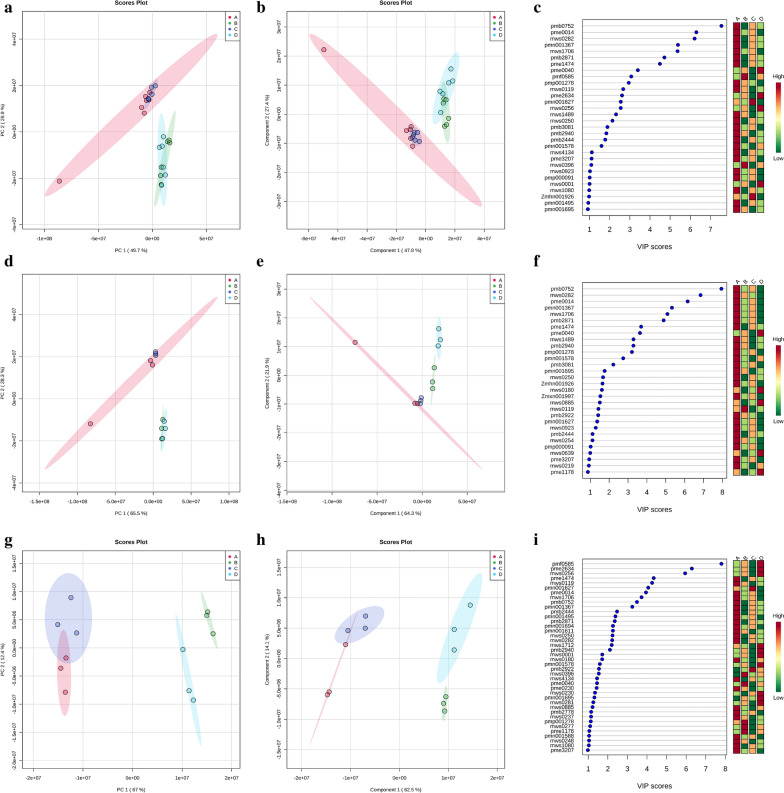
Multivariate analysis of the metabolites. PCA analysis of the metabolites for both varieties (**a**), PLS-DA analysis of the metabolites for both varieties (**b**), variable importance projection of PLS-DA comparing of the experimental treatments for both varieties (**c**). PCA analysis of the metabolites for Xiangyaxiangzhan (**d**), PLS-DA analysis of the metabolites for Xiangyaxiangzhan (**e**), Variable importance projection of PLS-DA comparing of the experimental treatments for Xianigyaxiangzhan (**f**). PCA analysis of the metabolites for Yuxiangyouzhan (**g**), PLS-DA analysis of the metabolites for Yuxiangyouzhan (**h**), Variable importance projection of PLS-DA comparing of the experimental treatments for Yuxiangyouzhan (**i**). **a** ZnO NPs0 + Cd 0, **b** ZnO NPs 0 + Cd 100, **c** ZnO NPs 50 + Cd 0, **d** ZnO NPs 50 + Cd 100

The shoot of both the rice varieties under the four treatments were constructed by PCA (Fig. 7a) and PLS-DA (Fig. 7b), considering all the metabolites. Two principal components together explained more than 75% of the variance. There were 26 metabolites with VIP values greater than 1, which could be considered as the main compounds to distinguish the treatment effects. Among the 26 metabolites, treatment A showed the highest values of 19 metabolites. Treatment B had the highest values of pmf0585 (dl-proline) and mws0396 (elaidic acid) whereas treatment C showed the highest values of pmn001627 (glucogallin). Treatment D had the highest values of pme0040 (adenine), pme2634 (dl-norvaline), mws0256 (l-valine), and mws0001 (l-asparagine anhydrous) (Fig. 7c, Additional file 3: Table S2).

The PCA and PLS-DA were constructed considering all the metabolites in the shoot of Xiangyaxiangzhan (Fig. 7d and e). Two principal components together explained more than 85% of the variance. There were 26 metabolites that had VIP values greater than 1, which could be considered as the main compounds that distinguish the treatments in Xiangyaxianzhan. Among the 26 metabolites, treatment A showed the highest values of 22 metabolites. Treatment B had the highest values of mws0119 (myristic acid), whereas treatment D resulted in the highest values of pme0040 (adenine), mws0180 (2,5-dihydroxybenzoic acid), and mws0885 (2,4-dihydroxy benzoic acid) (Fig. 7f, Additional file 3: Table S2).

The PCA and PLS-DA were constructed considering all the metabolites in the shoot of Yuxiangyouzhan (Fig. 7g and h). Two principal components together explained more than 75% of the variance. There were 40 metabolites that had VIP values greater than 1, which could be considered as the main compounds that distinguished the treatments for Yiangyaxianzhan. Among the 40 metabolites, treatment A showed the highest values of 23 metabolites, whereas treatment B had the highest values of mws0396 (elaidic acid), pme0040 (adenine), pmp001278 (lysoPC (16:0), mws0277 (kinic acid), and pme1178 (guanosine). Treatment *C* showed the highest values of pmn001627 (glucogallin) and pmb2922 (uridine 5'-diphospho-d-glucose), while treatment D resulted in the highest values of pmf0585 (dl-proline), pme2634 (dl-norvaline), mws0256 (l-valine), mws1712 (d-(+)-phenylalanine), pmb2940 (1-o-β-d-glucopyranosyl sinapate), mws0180 (2,5-dihydroxybenzoic acid), pmn001578 (hexadecanoic acid), mws0230 (l-(−)-threonine), pmn001695 (trihydroxycinnamoylquinic acid), and mws0281 (citric acid) (Fig. 7i, Additional file 3: Table S2).

**Fig. 8:**
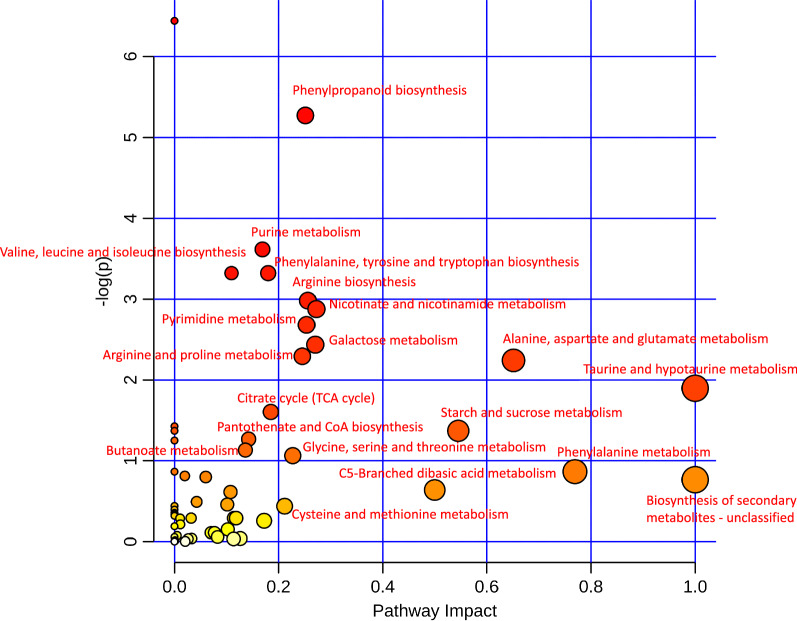
The pathways matched according to the p values and the pathway impact values

Moreover, enrichments in pathways such as ‘phenylpropanoid biosynthesis’, ‘purine metabolism’, ‘valine, leucine and isoleucine biosynthesis’, ‘phenylalanine, tyrosine and tryptophan biosynthesis’, ‘arginine biosynthesis’, ‘nicotinate and nicotinamide metabolism’, ‘pyrimidine metabolism’, ‘galactose metabolism’, ‘arginine and proline metabolism’, ‘alanine, aspartate and glutamate metabolism’, ‘taurine and hypotaurine metabolism’, ‘citrate cycle (TCA cycle)’, ‘starch and sucrose metabolism’, ‘pantothenate and CoA biosynthesis’, ‘butanoate metabolism’, ‘glycine, serine and threonine metabolism’, ‘phenylalanine metabolism’, ‘C5-branched dibasic acid metabolism’, ‘biosynthesis of secondary metabolites – unclassified’, and ‘cysteine and methionine metabolism’ were identified from the detected metabolites (Fig. 8).

#### Differential metabolites identified along with their involvement in metabolic processes

The PCA of metabolic profiles in shoot of rice seedling of Xiangyaxiangzhan and Yuxiangyouzhan under all experimental treatments were constructed (Additional file 1: Figure S4A). A total of 68, 7, 67, 33, and 78 significantly different metabolites were identified for X_A vs X_B, X_A vs X_C, X_A vs X_D, X_B vs X_D, and X_C vs X_D, respectively. A total of 55, 26, 42, 4, and 28 significantly different metabolites were identified for Y_A vs Y_B, Y_A vs Y_C, Y_A vs Y_D, Y_B vs Y_D, and Y_C vs Y_D, respectively. Moreover, 36, 5, 43, 9, and 18 downregulated metabolites and 32, 2, 24, 24, and 18 upregulated metabolites were identified for X_A vs X_B, X_A vs X_C, X_A vs X_D, X_B vs X_D, and X_C vs X_D, respectively, while 24, 22, 20, 2, and 6 downregulated metabolites and 31, 4, 22, 2, and 22 upregulated metabolites were identified for Y_A vs Y_B, Y_A vs Y_C, Y_A vs Y_D, Y_B vs Y_D, and Y_C vs Y_D, respectively (Additional file 1: Figure S4B, Additional file 4: Table S3).

The Venn diagram was constructed for the differential metabolites identified in the shoots after treatments for each variety (Additional file 1: Figure S4C). The KEGG enrichment analyses of the identified significantly different metabolites were assessed, and the ranking of the identified significantly differential metabolites in Xiangyaxiangzhan and Yuxiangyouzhan under different treatments was discerned (Additional file 1: Figure S4D and E). The metabolites such as Hmfn00531 (l-ascorbic acid), pme2563 (γ-glu-cys), pmb0874 [lysope 18:2(2n isomer)], pme3007 (uridine 5’-diphosphate), pme0040 (adenine), pme1474 (5'-deoxy-5'-(methylthio) adenosine), pmb0998 (guanosine 5'-monophosphate), and mws4134 (oxidized glutathione) had greater FC values at X_A vs X_B and Y_A vs Y_B. The metabolites of pme0256 (xanthine) with greater FC value were detected at X_A vs X_C and Y_A vs Y_C. The metabolites of Hmfn00531 (l-ascorbic acid), pme0256 (xanthine), pme2563 (γ-glu-cys), pme0040 (adenine), pmn001689 (9-Hydroxy-12-oxo-10-octadecenoic acid), pme3154 ((rs)-mevalonic acid), pme1474 (5'-deoxy-5'-(methylthio) adenosine), and mws4134 (oxidized glutathione) were identified with greater FC values for both X_A vs X_D and Y_A vs Y_D. Similar metabolites with greater FC value were not detected in case of X_B vs X_D and Y_B vs Y_D. While for X_C vs X_D and Y_C vs Y_D, the metabolites of pme0256 (xanthine), pme2563 (γ-glu-cys), pme0040 (adenine), pme0033 (hypoxanthine), pme3154 ((rs)-mevalonic acid), mws4134 (oxidized glutathione), pme1474 (5'-deoxy-5'-(methylthio) adenosine), pmp000260 (6-O-galloyl-β-d-glucopyranoside), pme1086 (glutathione reduced form) and pmb3066 (5-o-p-coumaroyl shikimic acid o-hexoside) were detected with greater FC values (Additional file 2: Figure S4E, Additional file 4: Table S3).

According to the KEGG enrichment analyses, ZnO NPs and Cd triggered metabolites were involved in metabolic pathways such as ‘zeatin biosynthesis’, ‘purine metabolism’, ‘pyrimidine metabolism’, ‘glutathione metabolism’, ‘cysteine and methionine metabolism’, ‘arginine and proline metabolism’, ‘biosynthesis of unsaturated fatty acids’, ‘caffeine metabolism’, and ‘flavonoid biosynthesis’/ ‘stilbenoid, diarylheptanoid and gingerol biosynthesis’, and the metabolites linking the pathways were assessed (Additional file 1: Figures S4D and S5).

Compared to ZnO NPs 0 + Cd 0 treatment, the ZnO NPs 0 + Cd 100 treatment caused inhibition of adenosine 5'-monophosphate, 5'-deoxy-5'-(methylthio) adenosine, guanosine 5'-monophosphate, cytidine 5'-monophosphate (cytidylic acid), and uridine 5'-monophosphate, and upregulated metabolites such as adenine, uridine 5’-diphosphate, adenine, xanthine, uridine 5’-diphosphate, γ-glu-cys, l-ascorbic acid, eicosenoic acid, eicosadienoic acid, and xanthine. The ZnO NPs 50 + Cd 0 treatment resulted in reduction in xanthine. Moreover, ZnO NPs 50 + Cd 100 treatment upregulated metabolites such as adenine, xanthine,γ-Glu-Cys, l-ascorbic acid, eicosenoic acid, and eicosadienoic acid, which led to inhibition in the metabolites such as 5'-deoxy-5'-(methylthio)adenosine and duanosine 5'-monophosphate (Additional file 1: Figure S4B-L, Additional file 5: Table S4). The other metabolites of the metabolic pathways varied for the varieties and the ZnO NPs treatment, with more metabolite-based changes in D/B and D/C for Xiangyaxiangzhan being detected than Yuxiangyouzhan (Additional file 5: Table S4). The (5-l-glutamyl)-l-amino acid in Yuxinagyouzhan was higher than Xiangyaxiangzhan (Additional file 5: Table S4).

## Discussion

Cd causes inhibitory effects on crop growth and such effects are generally concentration-dependent and genotype-specific. In this study, Cd treatment severely inhibited the seed germination of both rice varieties, which was consistent with previous study of Ahsan et al. [13] who reported that Cd toxicity alters the physiological and protein profiles in growing rice plants. The ZnO NPs treatment showed no notable effect on the seed germination of both rice varieties (Fig. 1a, b). In previous study, the effect of nanomaterials like polystyrene nanoplastics were found to have a prominent effect on seed germination and early growth of wheat [49]. In addition, Xiangyaxiangzhan and Yuxiangyouzhan are tolerant and sensitive to Cd, respectively as observed in our previous experiments (data unpublished), where changes in the dry weight of the shoot under Cd treatment were evaluated. However, we detected a higher germination rate in Xiangyaxiangzhan than Yuxinagyouzhan. Regarding seedling growth in terms of biomass accumulation, the Yuxiangyouzhan performed better than Xiangyaxiangzhan (Additional file 2: Table S1 and Fig. 2). The seed germination and seedling growth were varied for the two varieties under Cd toxicity.

Additionally, application of ZnO NPs mitigated the Cd toxicity on the growth of rice seedlings, promoting an increase in seedling weight, particularly in the shoot as well as the total fresh weight, while also promoting an increase in the root-shoot ratio (Fig. 2). However, the effect of all ZnO NPs treatments on the growth of rice seedling remained statistically similar (*p* > 0.05) (Fig. 2). Previous studies have reported the negative effect of Cd on the seed germination, root length, shoot, and biomass of rice seedlings [13, 21], however, the effect of ZnO NPs on the growth of rice remains controversial. For instance, the positive effect ZnO NPs at 50 ppm concentration on the growth of rice was noticed [29], whereas ZnO NPs treatment with 500 and 1000 ppm concentrations had negative effects on the early growth of rice. In contrast, 25, 50 and 100 mg L^− 1^ ZnO NPs treatments had a negative effect on the synthesis of chlorophylls and the growth of rice seedlings under hydroponic culture [32]. In this study, the ZnO NPs treatments changed the chl a, chl b, total chl content, and carotenoids in shoot of rice, but different varieties response differently (Additional file 1: Figure S2). So, exogenous application ZnO NPs within a suitable range/concentration would be able to promote the growth of rice, whereas high concentrations could have inhibitory effects.

Starch is an important storage polysaccharide for energy involved in the growth of rice seedlings. A previous study showed that α-amylases could facilitate both seed germination and seedling growth by mobilizing nutrients in the endosperm, however, starch would be immobilized under the Cd toxicity, which resulted in growth inhibition [20]. The exposure to Cd or ZnO NPs-based treatment had no significant effect on the α, β and total amylase activity (Fig. 3). The ZnO NPs treatment increased the α-amylase and total amylase activity under Cd stress (Fig. 3), which may be beneficial for the metabolism of stored substances in rice grains and may improve the early growth of rice.

Moreover, seed priming with ZnO NPs regulated the antioxidant enzyme activity i.e., SOD, POD, CAT, as well as MT and MDA contents in both rice varieties. Normally, SOD reduces O_2_^−^ to H_2_O_2_ and O_2_, whereas CAT scavenges the H_2_O_2_ generated during the photorespiration and β-oxidation of fatty acids [50]. The POD is located in cytosol, vacuole, cell wall, as well as in extracellular space, and uses guaiacol as an electron donor, while utilizing the H_2_O_2_ in the oxidation of various inorganic and organic substrates. Cd toxicity enhances the production levels of ROS, which increases lipid peroxidation, resulting in an increased generation of MDA as a byproduct [50, 51]. Besides, in order to mitigate the negative effect of ROS, the antioxidant enzyme activities increased at lower Cd concentrations but decreased at higher Cd levels [51–53]. In this study, Cd treatment reduced the SOD activity, but increased the POD and CAT activity, along with the MDA content in the root and shoot of the seedling (Figs. 4 and 5a, b). Previously, ZnO NPs treatment reduced the ROS generation, and induced higher activities of antioxidant enzymes, i.e., SOD, CAT and POD [29]. In this study, ZnO NPs treatment only increased the POD activity in shoot for Xiangyaxiangzhan (Fig. 4). Under Cd treatment, the ZnO NPs treatment had notable effects on the SOD, POD and CAT activity (Fig. 4). In addition to antioxidants, the MT is one of the most potent bioactive substances that scavenges ROS [54], and is utilized by plants to decrease the heavy metal concentration [55]. MT content in root was increased by the Cd treatment, while no significant increase was noted in shoot (Fig. 5c, d). A higher MT content in shoot of rice seedlings under ZnO NPs and Cd treatments was also noted (Fig. 5c, d). Overall, under Cd stress, seed priming with ZnO NPs treatment activated stress-resistance, while promoting the MT formation, and reducing the MDA accumulation in the plant.

The Cd accumulation in rice plant due to the competition between Zn and Cd ions as well as the dilution effect for improving plant biomass [56]. Study has confirmed the uptake of ZnO NPs in rice seedling when growing under hydroponic culture with ZnO NPs [32]. In this study, the concentration of soluble Zn in the seed priming solution was low (1.2–1.5 mg L^−1^) whilst the Zn concentration in the seed after priming were very high (up to 50 µg g^− 1^ DW) at ZnO NPs seed priming treatments (Additional file 1: Figure S1), which suggested that ZnO NPs was uptake by seeds during the seed priming period. Besides, the Zn concentration under ZnO NPs treatments is high for both varieties as compared to no ZnO NPs application, whilst the Cd level under ZnO NPs treatments reduced significantly for Xiangyaxingzhan but not for Yuxiangyouzhan (Fig. 6). Moreover, the ZnO NPs in the root and shoot tissue of the germinated seedlings may show positively associated with the Zn level increased in the seedlings which resulted from the transformation of the ZnO NPs from the seeds to the seedlings during seed germination (Additional file 1: Figure S3). Therefore, it was suggested that the ZnO NPs accumulation against Cd toxicity in plants, however the variety effect on the response of ZnO NPs treatments against the Cd toxicity should not be ignored.

Furthermore, ZnO NPs treatment at 50 mg L^−1^ showed substantial growth improvement of the rice seedlings under 100 mg L^− 1^ Cd stress. Therefore, the metabolites in the shoot of rice seedlings under the four treatments (A: ZnO NPs 0 + Cd, B: ZnO NPs 0 + Cd 100, C: ZnO NPs 50 + Cd 0, and D: ZnO NPs 50 + Cd 100) were assessed (Additional file 3: Table S2). Overall, there were 26 metabolites which had VIP values > 1, which could be considered as the main compounds for distinguishing the treatments in both varieties. For Xiangyaxiangzhan, there were 26 metabolites that had VIP values > 1, while there were 40 metabolites that had VIP values > 1 in Yuxiangyouzhan (Fig. 7). There were 20 important metabolic pathways identified in this study which were affected by the treatment and different varieties response to the pathways differently (Fig. 8 and Additional file 1: Figure S5). Regarding these pathways, most of them would be regulated under stress conditions in plants. For example, phenylpropanoid biosynthesis is important for plants response to biotic and abiotic environment [57]. Liu and Lin. [58] indicated that heat stress regulated the alanine, aspartate and glutamate metabolism in the leaves of *Sargassum fusiforme*. Hong et al. [59] suggested that genes involved in taurine and hypotaurine metabolism were affected by auxin, whereas above-mentioned pathways were possible important for rice response to ZnO NPs and Cd.

The regulations in glutathione metabolism in response to the Cd toxicity in this study are consistent with previously reports of Farooq et al. [60] who reported that modulations in ascorbate-glutathione cycle is associated with arsenic stress tolerance in oilseed rape. Moreover, amino acid-, purine-, carbon-, and glycerolipid-metabolic pathways were also affected by the Cd stress, which resulted in reduction of plant growth and/or photosynthetic capacity, and promoted the defense mechanisms to minimize cell damage [61]. The comparative analysis of the metabolites between the treatments with varied Cd and ZnO NPs doses allowed identification of metabolites regulating ‘zeatin biosynthesis’, ‘purine metabolism’, ‘pyrimidine metabolism’, ‘glutathione metabolism’, ‘cysteine and methionine metabolism’, ‘arginine and proline metabolism’, ‘biosynthesis of unsaturated fatty acids’, ‘caffeine metabolism’, and ‘flavonoid biosynthesis’/ ‘stilbenoid, diarylheptanoid and gingerol biosynthesis’ (Additional file 1: Figures S3 and S4). Among the treatments, ZnO NPs 0 + Cd 100 and ZnO NPs 50 + Cd 100 elicited pronounced change in metabolites, while the ZnO NPs 50 + Cd 0 showed marginal changes in metabolites. Changes in few other metabolites, such as (5-l-glutamyl)-l-amino acid was detected in both the rice varieties (Additional file 5: Table S4). The changes of these metabolites showed a consistent pattern with concomitant changes in growth and physiological parameters in response to the ZnO NPs under Cd stress.

Moreover, according to the analysis of the differential metabolites, high levels of L-Ascorbic acid, γ-glu-cys, and oxidized glutathione were detected in A vs B and A vs D for both varieties, while no significant change was observed in A vs C (Additional file 1: Figure S3E, Additional file 4: Table S3). Ascorbic acid (ASA) and glutathione (GSH) cycle are important pathways for a plant to cleanse the ROS [60]. The ascorbate peroxidase (APX) enzymes neutralize the H_2_O_2_ into H_2_O by utilizing ascorbate as an electron donor, which is subsequently oxidized to form monohydroascorbic acid (MDHA). The AsA-GSH cycle system may use AsA/DHA, GSH/GSSG, and NAD(P)H/NAD(P) to maintain an appropriate redox environment in plants [62, 63]. Besides, γ-glu-cys is utilized to synthesize GSH via the glutathione synthase pathway. The accumulation of γ-glu-cys is an important way for plant to protect itself under Cd stress [64]. Overall, seed priming with ZnO NPs treatment had a positive effect on the antioxidant enzymes of rice seedling under Cd stress. Notably, xanthine was detected in high concentrations in shoot of the rice seedling in A vs C, and A vs D for both rice varieties. Thus, the ZnO NPs treatment may affect the xanthine-related response pathways which are actively involved in the synthesis of DNA or RNA.

## Conclusions

In summary, seed priming with ZnO NPs had no obvious effects on the seed germination, however, it had substantially improved the seedling growth, owing to improvement in related physio-biochemical responses (such as activation of the amylase and the antioxidant enzymes), modulation in Zn concentration and ZnO NPs uptake in the seedling, and other metabolites in rice seedlings under Cd stress. Future in-depth of field studies are still needed to explore the exact mechanisms involved in ZnO NPs -induced improvements in early growth of rice under Cd toxic conditions. Overall, present findings may assist in developing feasible strategies for growing economically important crops in agricultural lands contaminated with high concentrations of heavy metals.

## Supplementary Information

**Additional file 1: Figure S1.** The change of pH and Zn^2+^ in the ZnO NPs solutions and the Zn concentration in the seed after priming. pH in the ZnO NPs solutions (A), Zn^2+^ in the ZnO NPs solutions (B), and the Zn concentration in the seed after priming (C). ZnO NPs 0, ZnO NPs 25, ZnO NPs 50 and ZnO NPs 100: 0 mg L^− 1^, 25 mg L^− 1^, 50 mg L^− 1^ and 100 mg L^− 1^ of ZnO NPs. Cd 0 and Cd 100: 0 mg L^− 1^ and 100 mg L^− 1^. Values were represented as mean ± SD (n = 4). Different low case letters among the treatments within a variety shows the statistically significant at *p* < 0.05 according to least significant different test.**Figure S2.** The chlorophyll and carotenoids content in shoot. The chlorophyll a content in shoot. (A), the chlorophyll b content in shoot. (B), the total chlorophyll content in shoot (C), and the carotenoids content in shoot (D). ZnO NPs 0, ZnO NPs 25, ZnO NPs 50 and ZnO NPs 100: 0 mg L^− 1^, 25 mg L^− 1^, 50 mg L^− 1^ and 100 mg L^− 1^ of ZnO NPs. Cd 0 and Cd 100: 0 mg L^− 1^ and 100 mg L^− 1^. Values were represented as mean ± SD (n = 4). Different low case letters among the treatments within a variety shows the statistically significant at *p* < 0.05 according to least significant different test.** Figure S3.** TEM images of the rice roots and shoot in germinated seedlings. Red arrows nanoparticles.**Figure S4.** Analysis of the metabolic profiles in shoot of rice seedling. Principal component analysis (PCA) of metabolic profiles in shoot of rice seedling of Xiangyaxiangzhan and Yuxiangyouzhan under control and treatments (A). The Identified total significant different metabolites and up- and down-regulated metabolites (B). The Venn diagram of the significant different metabolites among the treatments (C). KEGG enrichment analyses of the identified significant different metabolites (D) and ranking of the identified significant differential metabolites (E) in Xiangyaxiangzhan and Yuxiangyouzhan under different treatments. X_A: ZnO NPs 0 + Cd 0 for Xiangyaxiangzhan, X_B: ZnO NPs 0 + Cd 100 for Xiangyaxiangzhan, X_C: ZnO NPs 50 + Cd 0 for Xiangyaxiangzhan, X_D: ZnO NPs 50 + Cd 100 for Xiangyaxiangzhan; Y_A: ZnO NPs 0 + Cd 0 for Yuxiangyouzhan, Y_B: ZnO NPs 0 + Cd 100 for Yuxiangyouzhan, Y_C: ZnO NPs 50 + Cd 0 for Yuxiangyouzhan, Y_D: ZnO NPs 50 + Cd 100 for Yuxiangyouzhan. The abscissa indicates that the rich factor, ordinate corresponding to each pathway is the path name, and the color of the point is *p*-value, the redder the enrichment is more significant. The size of the points represents the number of enriched differential metabolites.**Figure S5.** Metabolic pathways network.

**Additional file 2: Table S1.** ANOVA analysis of the growth and physiological parameters.

**Additional file 3:Table S2.** Metabolites detected in the rice varieties.

**Additional file 4:Table S3.** The identified significant different metabolites in Xiangyaxiangzhan and Yuxiangyouzhan under different treatments.

**Additional file 5:Table S4.** Fold change of the detected metabolites involved in the metabolic pathways.

## Data Availability

The datasets used and/or analysed during the current study are available from the corresponding authors on reasonable request.
