# Supplementary material for: ZnO nanoparticle-based seed priming modulates early growth and enhances physio-biochemical and metabolic profiles of fragrant rice against cadmium toxicity
Source: J Nanobiotechnology. 2021 Mar 17;19:75. doi: 10.1186/s12951-021-00820-9 (PMC7968244; doi:10.1186/s12951-021-00820-9)
Supplement: Supplementary file 1 — Additional file 1: Figure S1. The change of pH and Zn2+ in the ZnO NPs solutions and the Zn concentration in the seed after priming. pH in the ZnO NPs solutions (A), Zn2+ in the ZnO NPs solutions (B), and the Zn concentration in the seed after priming (C). ZnO NPs 0, ZnO NPs 25, ZnO NPs 50 and ZnO NPs 100: 0 mg L− 1, 25 mg L− 1, 50 mg L− 1 and 100 mg L− 1 of ZnO NPs. Cd 0 and Cd 100: 0 mg L− 1 and 100 mg L− 1. Values were represented as mean ± SD (n = 4). Different low case letters among the treatments within a variety shows the statistically significant at p < 0.05 according to least significant different test.Figure S2. The chlorophyll and carotenoids content in shoot. The chlorophyll a content in shoot. (A), the chlorophyll b content in shoot. (B), the total chlorophyll content in shoot (C), and the carotenoids content in shoot (D). ZnO NPs 0, ZnO NPs 25, ZnO NPs 50 and ZnO NPs 100: 0 mg L− 1, 25 mg L− 1, 50 mg L− 1 and 100 mg L− 1 of ZnO NPs. Cd 0 and Cd 100: 0 mg L− 1 and 100 mg L− 1. Values were represented as mean ± SD (n = 4). Different low case letters among the treatments within a variety shows the statistically significant at p < 0.05 according to least significant different test. Figure S3. TEM images of the rice roots and shoot in germinated seedlings. Red arrows nanoparticles.Figure S4. Analysis of the metabolic profiles in shoot of rice seedling. Principal component analysis (PCA) of metabolic profiles in shoot of rice seedling of Xiangyaxiangzhan and Yuxiangyouzhan under control and treatments (A). The Identified total significant different metabolites and up- and down-regulated metabolites (B). The Venn diagram of the significant different metabolites among the treatments (C). KEGG enrichment analyses of the identified significant different metabolites (D) and ranking of the identified significant differential metabolites (E) in Xiangyaxiangzhan and Yuxiangyouzhan under different treatments. X_A: ZnO NPs 0 + Cd 0 for Xiangyaxiangzhan, X_B: ZnO NPs 0 [file 12951_2021_820_MOESM1_ESM.docx]

Fig. S1 The change of pH and Zn^2+^ in the ZnO NPs solutions and the Zn concentration in the seed after priming.

pH in the ZnO NPs solutions (A), Zn^2+^ in the ZnO NPs solutions (B), and the Zn concentration in the seed after priming (C). ZnO NPs 0, ZnO NPs 25, ZnO NPs 50 and ZnO NPs 100: 0 mg L^-1^, 25 mg L^-1^, 50 mg L^-1^ and 100 mg L^-1^ of ZnO NPs. Cd 0 and Cd 100: 0 mg L^-1^ and 100 mg L^-1^. Values were represented as mean ± SD (n = 4). Different low case letters among the treatments within a variety shows the statistically significant at *p* < 0.05 according to least significant different test.

Fig. S2 The chlorophyll and carotenoids content in shoot.

The chlorophyll a content in shoot. (A), the chlorophyll b content in shoot. (B), the total chlorophyll content in shoot (C), and the carotenoids content in shoot (D). ZnO NPs 0, ZnO NPs 25, ZnO NPs 50 and ZnO NPs 100: 0 mg L^-1^, 25 mg L^-1^, 50 mg L^-1^ and 100 mg L^-1^ of ZnO NPs. Cd 0 and Cd 100: 0 mg L^-1^ and 100 mg L^-1^. Values were represented as mean ± SD (n = 4). Different low case letters among the treatments within a variety shows the statistically significant at *p* < 0.05 according to least significant different test.

Fig. S3 TEM images and DES images of the rice roots and shoot in germinated seedlings.

TEM images of the rice roots and shoot in germinated seedlings (A). Red arrows nanoparticles. DES images of the rice roots and shoot in germinated seedlings (B).

Fig. S4 Analysis of the metabolic profiles in shoot of rice seedling.

Principal component analysis (PCA) of metabolic profiles in shoot of rice seedling of Xiangyaxiangzhan and Yuxiangyouzhan under control and treatments (A). The Identified total significant different metabolites and up- and down-regulated metabolites (B). The Venn diagram of the significant different metabolites among the treatments (C). KEGG enrichment analyses of the identified significant different metabolites (D) and ranking of the identified significant differential metabolites (E) in Xiangyaxiangzhan and Yuxiangyouzhan under different treatments.

X_A: ZnO NPs 0 + Cd 0 for Xiangyaxiangzhan, X_B: ZnO NPs 0 + Cd 100 for Xiangyaxiangzhan, X_C: ZnO NPs 50+Cd 0 for Xiangyaxiangzhan, X_D: ZnO NPs 50 + Cd 100 for Xiangyaxiangzhan; Y_A: ZnO NPs 0 + Cd 0 for Yuxiangyouzhan, Y_B: ZnO NPs 0 + Cd 100 for Yuxiangyouzhan, Y_C: ZnO NPs 50 + Cd 0 for Yuxiangyouzhan, Y_D: ZnO NPs 50 + Cd 100 for Yuxiangyouzhan. The abscissa indicates that the rich factor, ordinate corresponding to each pathway is the path name, and the color of the point is *p*-value, the redder the enrichment is more significant. The size of the points represents the number of enriched differential metabolites.

Fig. S5 Metabolic pathways network.

The metabolites with similar color with the pathways mean the linking metabolites between two pathways. A: ZnO NPs 0 + Cd 0, B: ZnO NPs 0 + Cd 100, C: ZnO NPs 50 + Cd 0, D: ZnO NPs 50 + Cd 100. Fold changes of metabolites in red color indicated significant up-regulation (fold change > 2) and in green color showed significant down-regulation (fold change < 0.5), in comparison with normalized metabolite concentrations under treatment and control condition; and in white color indicated no significant changes. Metabolic pathways were constructed on basis of KEGG database (http://www.genome.jp/kegg/).
