# Supplementary material for: ZnO nanoparticle-based seed priming modulates early growth and enhances physio-biochemical and metabolic profiles of fragrant rice against cadmium toxicity
Source: J Nanobiotechnology. 2021 Mar 17;19:75. doi: 10.1186/s12951-021-00820-9 (PMC7968244; doi:10.1186/s12951-021-00820-9)
Supplement: Supplementary file 2 — Additional file 2: Table S1. ANOVA analysis of the growth and physiological parameters. [file 12951_2021_820_MOESM2_ESM.docx]

Table S1 ANOVA analysis of the growth and physiological parameters.

| Parameter | V | ZnO NPs | Cd | V×ZnO NPs | V×Cd | ZnO NPs×Cd | V×ZnO NPs×Cd |
| --- | --- | --- | --- | --- | --- | --- | --- |
| Germination | ** | ns | * | ns | ns | ns | ns |
| Seed vigor index | ns | ns | ** | ns | ns | ns | * |
| Total fresh weight | ** | ** | ** | ** | ns | ** | ** |
| Shoot fresh weight | ** | ** | ** | ns | ** | ** | ns |
| Root fresh weight | ** | ** | ** | ** | ** | ** | ** |
| Shoot length | * | ** | ** | ns | * | ** | ns |
| Root length | * | ** | ** | ns | ns | ** | ns |
| Prophyll leaf length | * | ** | ** | ** | * | ** | ** |
| Leaf sheath length | * | ** | ** | ** | * | ** | * |
| Leaf blade length | ns | ** | ** | * | * | ** | * |
| α-amylase activity in shoot | ** | ** | ** | ** | ** | ** | ns |
| α-amylase activity in the seedling | ns | ** | ns | ns | ** | ** | ** |
| β-amylase activity in shoot | * | ns | ns | ns | ns | * | ** |
| β-amylase activity in the seedling | ns | ns | ns | * | ns | ns | ns |
| Total amylase activity in shoot | * | ** | ** | ** | ** | ns | ** |
| Total amylase activity in the seedling | ns | ** | ns | ns | ** | ** | * |
| SOD activity in shoot | * | ** | ** | ** | ** | ns | ** |
| SOD activity in the seedling | * | ** | ** | ns | * | ns | ns |
| POD activity in shoot | ** | ** | ** | ** | ** | ** | ** |
| POD activity in the seedling | ns | * | ** | ns | ns | ** | ** |
| CAT activity in shoot | ** | ** | ** | ** | ** | ** | ** |
| CAT activity in the seedling | ** | ** | ** | ** | ** | ** | ** |
| MDA content in shoot | * | ** | ** | ns | ns | ns | ** |
| MDA content in the seedling | ** | ** | ** | ** | * | ** | * |
| MT concentration in shoot | * | ** | * | ** | ns | ** | ** |
| MT concentration in the seedling | ** | ** | ** | ** | ns | ** | * |
| Chlorophyll a content in shoot | ** | ** | ** | ** | ** | ** | ns |
| Chlorophyll b content in shoot | ** | ** | ** | ** | ** | ** | ns |
| Total chlorophyll content in shoot | ** | ** | ** | ** | ** | ** | ns |
| Carotenoids content in shoot | ** | ** | ** | ** | ** | ** | ns |
| Zn concentration in the seedling | * | ** | ns | ** | ns | ** | ** |
| Cd concentration in the seedling | * | ** | ** | ** | ns | ** | ** |

V: variety. Cd: Cd treatment. ZnO NPs: ZnO nanoparticles treatment. *, significant at *p* < 0.05; **, significant at *p* < 0.01; ns, nonsignificant.
